# Comparison of Anti-Renal Fibrosis Activity of *Eucommiae cortex* Extract and Its Microbial Fermentation Products

**DOI:** 10.3390/ph18050747

**Published:** 2025-05-19

**Authors:** Zhengyou He, Wenyi Jiang, Ruijiao Yao, Wenyan Xiao, Zhiyang Chen, Miao Zheng, Xia Zeng, Jia Li, Zhengwen Li, Yong Jiang

**Affiliations:** Laboratory of Chinese Medicine Resources and New Product Development, College of Pharmacy, College of Food and Bioengineering, Chengdu University, Chengdu 610106, China

**Keywords:** chronic kidney disease (CKD), *Eucommiae cortex* (EC), fermentation, TGF-β1/Smad, renal fibrosis

## Abstract

**Background:** Renal fibrosis is a common pathological feature of all progressive chronic kidney disease (CKD). *Eucommiae cortex* (EC) is a valuable economic tree species endemic to China. The microbial fermentation of Chinese medicines can release their active ingredients as effectively as possible or produce new active ingredients with enhanced efficacy and reduced toxic side effects; **Methods:** The microbial fermentation of EC can produce pinoresinol (Pin) and dehydrodiconiferyl alcohol (DA). In this study, C57 BL/6 mice were fed a diet containing 0.2% adenine, resulting in a model of chronic kidney disease. The effects of EC and EC ferment (ECF) on CKD were explored by the exogenous supplementation of EC and ECF; **Results:** The results of the study showed that exogenous supplementation with EC and ECF suc-cessfully reduced creatinine and urea nitrogen levels, down-regulated the expression levels of TGF-β1, α-SMA, Smad3, and phospho-Smad3 in the TGF-β1/Smad signaling pathway, and ameliorated renal fibrosis; **Conclusions:** Both EC and ECF may have reno-protective effects and provide a reference for relevant clinical drug development.

## 1. Introduction

Renal fibrosis is a common pathophysiological feature of almost all progressive chronic kidney disease (CKD) [1]. Interstitial myofibroblasts are the primary effector cells in renal fibrosis, generating significant amounts of extracellular matrix (ECM), resulting in renal function loss and the concurrent degradation of renal structure [2]. The pathogenesis of renal fibrosis is intricate and complex, and experimental studies of Chinese medicines against renal fibrosis have made some progress, revealing the great potential of Chinese drugs in the prevention and treatment of renal fibrosis [3,4]. Numerous kidney diseases have been linked to the TGF-β1/Smad pathway. The process of renal fibrosis can be stopped and ECM deposition in renal tissues reversed by blocking the pathway’s activation [5]. Contemporary pharmacological research has shown numerous herbal remedies and monomer compounds to have antifibrotic properties. Natural medicine and its extracts may affect fibrosis by influencing the TGF-β1/Smad signal transduction pathway [6].

*Eucommiae cortex* (EC) is the dried bark of *Eucommia ulmoides*, a plant of the family Eucommiaceae [7], a precious economic tree species endemic to China. It is distributed in Sichuan, Guizhou, Yunnan, and other places and has a long history of medicinal use. It is now widely used in medicine, food processing, feed, industrial production, and other fields [8]. EC is rich in natural active ingredients, from which 246 compounds have been isolated and identified [9], mainly including lignans, iridoids, flavonoids, phenylpropanoids, and EC gum. Its pharmacological effects mainly include lowering blood pressure, enhancing immunity, regulating blood lipids, lowering blood sugar, protecting the liver, choleretic and diuretic activities, protecting nerve cells, regulating bone metabolism, tonifying and protecting the kidneys, and stabilizing the fetus [10].

EC has the effect of tonifying the liver and kidneys and strengthening muscles and bones. The lignans, iridoids, and phenylpropanoid constituents in EC are the main constituents and are the basis of medicinal substances in EC, which have a good anti-renal fibrosis effect [11]. The lignans in EC are protective against hypertensive renal damage, which may be related to inhibiting renal aldose reductase [12]. EC ameliorates hypertensive renal damage, diabetic nephropathy, and adenine renal injury-induced renal fibrosis in rats [13]. EC upregulates the expression of MMP-2 in the renal tissues of rats with unilateral ureteral obstruction (UUO), which slows down the disease progression of renal fibrosis [14]. EC’s decoction can improve patients’ renal function [15]. EC can inhibit the up-regulation of CTGF expression in the renal tubular epithelium of UUO rats, slowing the progression of tubular injury and interstitial fibrosis [16].

The microbial fermentation of traditional Chinese medicine has a long history. Microbial fermentation can release the active ingredients as soon as possible or produce new active ingredients with enhanced efficacy and reduced toxic side effects [17]. The microbial fermentation of EC can produce pinoresinol (Pin) and dehydrodiconiferyl alcohol (DA) [18]. Therefore, we studied the impact of EC and its microbial fermentation products on the TGF-β1/Smad pathway and renal fibrosis to inform clinical drug development.

## 2. Results

### 2.1. HPLC Results

**Figure 1:**
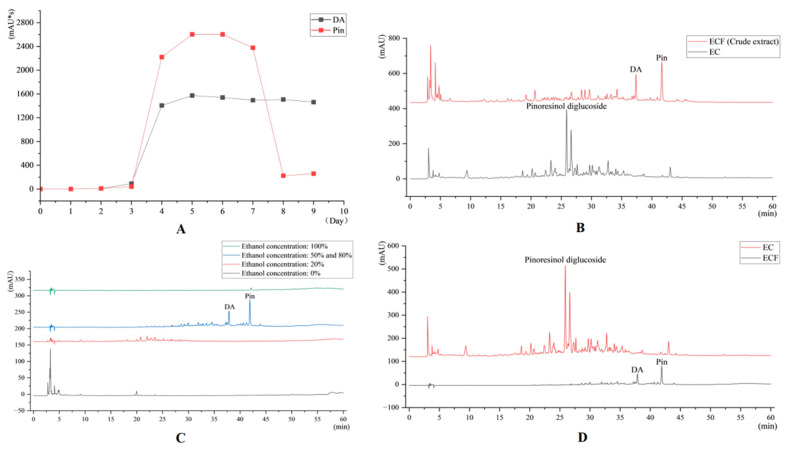
(**A**) Variation in Pin and DA content on different fermentation days. (**B**) HPLC results for EC and ECF crude extracts. (**C**) HPLC results for ECF crude extract eluate. (**D**) HPLC results for drug of EC and ECF groups.

The temporal profile of Pin and DA content during fermentation was analyzed (Figure 1A), and the highest content of both compounds was observed on the fifth day of fermentation. A comparative high-performance liquid chromatography (HPLC) analysis between EC extract and ECF crude extract was performed (Figure 1B). Different concentrations of eluent were collected, and based on the HPLC results, 50% ethanol eluent and 80% ethanol eluent were mixed, concentrated, and dried to obtain the final ECF drug formulation. The HPLC results of different concentration eluents of the EC crude extracts are shown in Figure 1C. The HPLC results of gavage preparation in the EC and ECF groups are shown in Figure 1D.

### 2.2. Effect of EC and ECF on Body Weight of Mice

**Figure 2:**
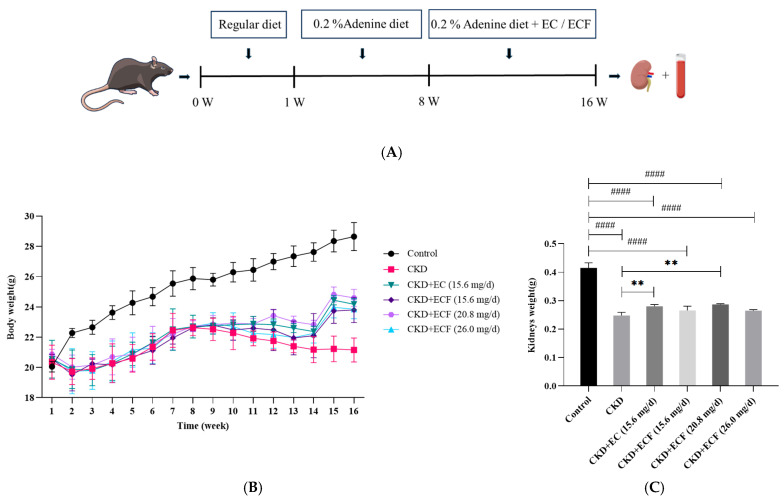
(**A**) Schematic diagram of the animal experiment. The chronic kidney disease model was established by feeding C57BL/6 mice a diet containing 0.2% adenine. (**B**) Body weight changes in different groups of mice at different times. (**C**) Comparative results of the weight of kidney tissues of different groups of mice. #### *p* < 0.0001 vs. control group; ** *p* < 0.01 vs. CKD group.

The experimental protocol for the animal model is shown in Figure 2A. The CKD group exhibited the lowest body mass and the control group maintained the highest during the study period (Figure 2B). Statistical analysis demonstrated that the control group showed significantly higher body weight compared to other groups on the second week post-modeling. By the 15th week, the CKD group displayed considerably reduced body weight relative to all other groups. A comparative analysis of kidney tissue weights (Figure 2C) showed that the control group had significantly higher kidney weights, while the CKD group had significantly lower kidney weights. These results suggest that EC and ECF can improve weight loss and reduce renal tissue damage caused by adenine-induced renal fibrosis.

### 2.3. Effects of EC and ECF on Creatinine and Urea Nitrogen in Mice

**Figure 3:**
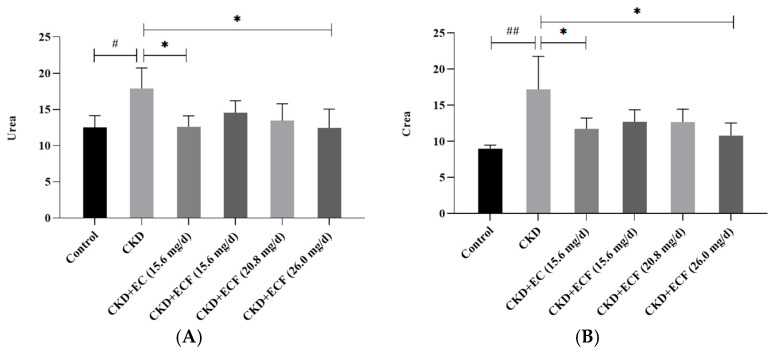
(**A**) Urea results. (**B**) Crea results. # *p* < 0.05, ## *p* < 0.01 vs. control group; * *p* < 0.05 vs. CKD group.

The mice in the CKD group exhibited significantly elevated serum creatinine (Crea) and urea nitrogen (Urea) concentrations compared to the control group. Remarkably, both EC and ECF treatments attenuated these abnormalities, with serum Urea and Crea levels in the EC/ECF-treated groups observed to approximate those of the control cohort (Figure 3A,B). The CKD + ECF (26 mg/day) group demonstrated the most pronounced reduction in Urea and Crea. The findings showed that EC and ECF may effectively decrease CKD mice’s Urea and Crea serum levels.

### 2.4. Effects of EC and ECF on Histopathologic Changes in Mice with Chronic Kidney Disease

**Figure 4:**
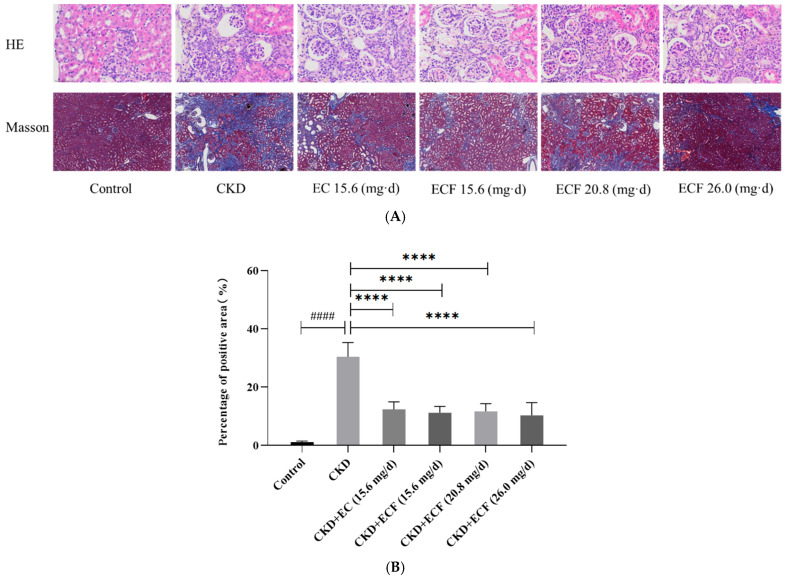
(**A**) HE and Masson staining results (HE: 400×; Masson: 40×). (**B**) Significance analysis of the area occupied by positive renal fibrosis. #### *p* < 0.0001 vs. control group; **** *p* < 0.0001 vs. CKD group.

The results of the Masson and HE staining of renal tissue are displayed in Figure 4A. All groups except the control group had tubular proteins, brownish-yellow material, foci of mineralization, and varied degrees of peritubular depressions and interstitial fibrosis. The samples from each group varied in terms of the degree of mineralization: control group < CKD + EC (15.6 mg/d) group < CKD + ECF (20.8 mg/d) group < CKD + ECF (26 mg/d) group < CKD + ECF (15.6 mg/d) group < CKD group. The fibrosis severity of the samples in each group was as follows: control group < CKD + ECF (26 mg/d) group < CKD + ECF (15.6 mg/d) group < CKD + ECF (20.8 mg/d) group < CKD + EC (15.6 mg/d) group < CKD group. The visual field’s blue area per unit area was measured, and the significance analysis of the percentage area of positivity in each group is shown in the graph (Figure 4B). There was a significant decrease in all other groups compared to the CKD group. The results indicated that EC and ECF attenuated adenine-induced renal fibrosis.

### 2.5. qRT-PCR Measurement of TGF-β1, Smad3, α -SMA, and mRNA Expression

**Figure 5:**
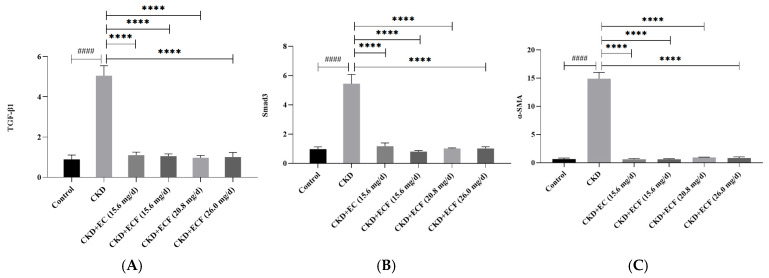
(**A**) TGF-β1 expression levels. (**B**) Smad3 expression levels. (**C**) α-SMA expression levels. #### *p* < 0.0001 vs. control group; **** *p* < 0.0001 vs. CKD group.

A relative quantitative analysis of target genes was performed using the control group to examine changes in the mRNA expression levels of target genes in kidney tissues compared to the control group. The TGF-β1, Smad3, and α-SMA expression levels were higher in the CKD group compared to the control group. As illustrated in Figure 5A–C, no significant differences were observed in TGF-β1, Smad3, and α-SMA expression levels between the EC, ECF, and control groups. The group with CKD + ECF (20.8 mg/d) had a more significant down-regulation of TGF-β1, while the group with CKD + ECF (15.6 mg/d) had a more substantial down-regulation of α-SMA and Smad3. The findings suggest that ECF and EC can reduce the expression of TGF-β1, Smad3, and α-SMA and attenuate the adenine-induced renal fibrosis.

### 2.6. Immunohistochemical Results

**Figure 6:**
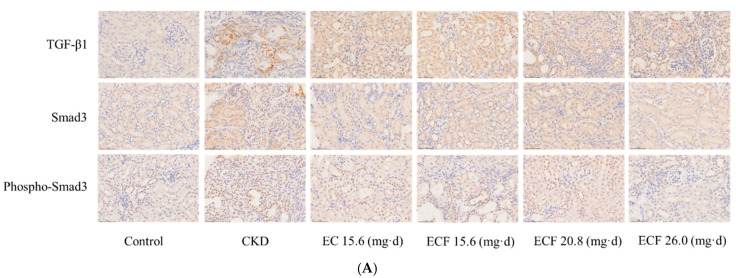

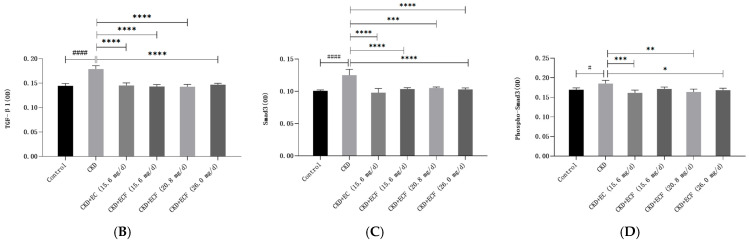
(**A**) Immunohistochemical results (400×). (**B**) TGF-β1 expression levels. (**C**) Smad3 expression levels. (**D**) Phospho-Smad3 expression levels. # *p* < 0.05, #### *p* < 0.0001 vs. control group; * *p* < 0.05, ** *p* < 0.01, *** *p* < 0.001, **** *p* < 0.0001 vs. CKD group.

Each group’s renal tissues underwent immunohistochemical staining. The average optical density (OD) of the positive expression unit field of view was measured, and the results are shown in Figure 6A. The TGF-β1, Smad3, and phospho-Smad3 levels were considerably more significant in the CKD group than in the control group. They were significantly lower in the EC and ECF groups than in the CKD group, as illustrated in Figure 6B–D. TGF-β1 was considerably down-regulated in the CKD + ECF group (20.8 mg/d); Smad3 and phospho-Smad3 were significantly down-regulated in the CKD + EC group (15.6 mg/d). The findings showed that in the renal tissues of CKD mice, the ECF and EC groups dramatically decreased the expressions of TGF-β1, Smad3, and phospho-Smad3.

## 3. Discussion

Microbial fermentation can change the content and structure of existing compounds in plants, reduce toxic substances, and produce abundant secondary metabolites through various metabolic pathways [19]. For example, microbial fermentation promotes the release of active components such as phenolic compounds and flavonoids [20], and antioxidant activity is improved [21]. Combining fermentation with EC may lead to more possibilities. Focusing on the investigation and optimization of the fermentation performance of different oil-containing yeasts on *Eucommia hydrolysate*, the aim was to explore and evaluate the functions and effects of fermentation with mixed strains compared to single strains [22]. The degradation patterns of hemicellulose, cellulose, and lignin in raw materials by solid-state fermentation with three edible fungi were investigated using *Eucommia bark* and *Eucommia leaf* residue as substrates [23]. We studied the fermentation of *Cordyceps militarisin* order to increase the content of active ingredients in Eucommia and poplar flowers and then utilize the nutrients to promote the growth of *Cordyceps militaris* and increase the production of antibacterial and antiviral actives [24]. To explore the effect of probiotic fermentation on some active ingredients of EC, Mulberry leaves, and Gynostemma, the author used three mixed probiotic bacteria, including Bacillus subtilis, to start fermentation tests on three herbal decoctions and their decoction mixtures, respectively [25]. A kind of lactic acid bacteria was also used to ferment EC mushroom sauce [26]. EC is fermented by microorganisms to produce the fermentation products Pin and DA, so this experiment focused on the mitigating effects of EC and ECF on renal fibrosis.

The results of the study showed that exogenous supplementation with EC and ECF successfully reduced creatinine and urea nitrogen levels; down-regulated the expression levels of TGF-β1, α-SMA, Smad3, and phospho-Smad3 in the TGF-β1/Smad signaling pathway; and ameliorated renal fibrosis. As a result, both EC and ECF may have renoprotective effects and provide a reference for relevant clinical drug development. However, the underlying process of CKD is complex and includes many cytokines and signaling pathways. Whether ECF and EC can ameliorate CKD through other signaling pathways needs further exploration.

## 4. Materials and Methods

### 4.1. Reagents and Antibodies

Specific information is provided in Table 1. EC was purchased from Sichuan Zangxi tang Biotechnology Co., Ltd. (No. 230625, Guanghan, China); chemicals and HPLC-grade acetonitrile were purchased from Chengdu kelong chemical Co., Ltd. (Chengdu, China).

### 4.2. EC Extraction and Drug Preparation

The EC powder was subjected to methanol-assisted thermal reflux extraction under optimized conditions, with a material-to-solvent ratio of 1:20 (*w*/*v*). After ultrasonic extraction for 30 min, the filtrate was taken and the residue was extracted again using the above method, and then the two filtrates were combined and vacuum-filtrated through Whatman No. 1 filter paper. The pooled filtrates were concentrated via rotary evaporation (Yingyu R201D system, 40 °C, 0.09 MPa vacuum) and subsequently vacuum-dried to yield the EC extract. The EC extract was dissolved in methanol, analyzed by HPLC, and compared with the relevant regulations of EC in the *Chinese Pharmacopoeia*. Batches of acceptable quality were taken for subsequent experiments. The EC extract was dissolved in 0.05% carboxymethylcellulose sodium (CMC-Na) to make a suspension for gastrointestinal administration according to the EC dose recommended by the *Chinese Pharmacopoeia* and referring to the literature dose [27].

### 4.3. Microbial Fermentation Product Preparation

#### 4.3.1. Optimal Fermentation Time

The fermentation broth containing EC extract was configured. Here, 1000 mL of fermentation solution contained 200 g of fresh potato, 20 g of analytically pure glucose, 1.5 g of analytically pure magnesium sulfate, 3 g of analytically pure potassium dihydrogen phosphate, and 140 mg of VB_1_. The EC extract was obtained by extracting 50 g of EC powder according to the method described in Section 4.2. The fermentation process employs traditional mucor [18] for microbial transformation. Fermentation medium aliquots (10 mL) were aseptically sampled at 24 h intervals (Days 1–9). Methanol (1:2 *v*/*v*) was added to the sample and sonicated (60 min). The solution was concentrated to near dryness using a rotary evaporator, dissolved in anhydrous methanol (AR grade), and filtered through a 0.22 μm nylon membrane before HPLC analysis.

#### 4.3.2. Macroporous Resin Fractionation

The EC fermentation broth with optimal fermentation time was screened according to the HPLC results of different fermentation times. The fermentation broth was taken and added with methanol (1:2 *v*/*v*) and ultrasonicated (60 min). Then, the solution was concentrated to near dryness using a rotary evaporator and dried in a reduced-pressure drying oven to obtain the crude extract of ECF. AB-8 resin was pre-conditioned through sequential ethanol (95%, *v*/*v*) and deionized water (18.2 MΩ·cm) washing cycles. The ECF crude extract was loaded onto the adsorbent at 3 mL/min, and then eluted with ethanol using a stepwise gradient: 0% → 20% → 50% → 80% →100% at 2 mL/min under UV monitoring (280 nm). The eluted fractions were rotary-evaporated, lyophilized, and analyzed by HPLC.

#### 4.3.3. ECF Drug Preparation

The ECF crude extract product of microbial fermentation of EC was taken at the optimum fermentation time. Gradient ethanol elution was performed using AB-8 macroporous adsorbent resin. The target fractions were collected, concentrated under reduced pressure (40 °C, 0.09 MPa), and lyophilized to obtain the ECF extract. Based on the daily dosage of EC specified in the *Chinese Pharmacopoeia*, the lyophilized extract was homogenized with 0.05% CMC-Na [28] to formulate a gastric suspension in the recommended dose.

### 4.4. Experimental Animals

Male C57BL/6 mice (21 ± 2 g body weight) were procured from Chengdu Dashuo Biotechnology Co., Ltd. (Animal Production License No. SCXK 200-030; Chengdu, China). The animal experimental protocols and operational procedures were based on national regulations on experimental animal welfare and ethics. They were examined by the Animal Welfare Ethics Committee of Chengdu Medical College (No. 014, Cheng Yi Dong Lun [2022]).

### 4.5. HPLC Conditions

Chromatographic conditions: a Unisil 5-120 C18 ultra column (5 μm, 4.6 mm × 250 mm), temperature maintained at 30 °C, with a mobile phase flow rate of 1 mL/min and detection wavelength at 227 nm. Detailed elution parameters are provided in Table 2.

### 4.6. Adenine-Diet-Induced Renal Fibrosis

After a week of acclimatization feeding, 36 male C57BL/6 mice (22 ± 2 g) were randomly divided into six groups (n = 6): control, CKD, CKD + EC (15.6 mg/d), CKD + ECF (15.6 mg/d), CKD + ECF (20.8 mg/d), and CKD + ECF (26 mg/d). Except for the control group, which was given a typical control diet (Wuxi Daitz Biotechnology Co., Ltd.), all groups were provided meals containing 0.2% adenine [29]. The blank and CKD groups were gavaged with 0.2 mL of CMC-Na solution daily, while the CKD + EC and CKD + ECF groups were gavaged once daily with 0.2 mL of 0.05% CMC-Na solution containing the relevant concentration of the drug. After 16 weeks, the mice were euthanized, and samples of blood and kidney tissue were gathered for additional research.

### 4.7. Renal Function Analysis

The blood samples were placed in an incubator at 37 °C for 30 min and then transferred to a centrifuge that had been pre-cooled to 4 °C. The supernatant was extracted for kidney function measurements following a 15 min [30] centrifugation at 3000 rpm. A fully automated biochemical analyzer (model: 3100) was used to measure renal function parameters such as Crea and Urea.

### 4.8. Analysis of Renal Histopathology

HE: For embedding, 4% paraformaldehyde-fixed tissue samples were rinsed under running water, trimmed, and placed into pathological embedding plastic baskets for gradient alcohol dehydration, xylene transparency, wax dipping, and embedding. Sectioning: tissues were cut into 5 µm thick slices using a Leica RM2235 slicer, spread in warm water, and fixed onto slides. Staining: sections were deparaffinized to water, stained with hematoxylin–eosin, dehydrated in gradient alcohol, made transparent using xylene, and sealed with neutral resin glue. Pathological changes in the liver and kidney were observed under the microscope. The entire sectioned area was observed, and photographic recordings were made using a microimaging system for each group of areas with apparent lesions. Masson staining: Sections were deparaffinized to water and stained with Weigert’s hematoxylin stain, Lichun red acidic magenta solution, 1% phosphomolybdic acid, 1% glacial acetic acid, aniline blue staining solution, and gradient alcohol dehydration followed by xylene transparency, and the sections were sealed with neutral resin glue. Renal tissues were photographed with a microimaging system. The area of positive expression in the pictures was measured using Image-Pro plus 6.0.

### 4.9. qRT-PCR Measurement of TGF-β1, Smad3, and α-SMA mRNA Expression

#### 4.9.1. RNA Extraction and Concentration Determination

The procedure is as follows: Take an appropriate amount (about 100 mg) of tissue sample, add 1 mL of RNAiso Plus, homogenize, and leave at room temperature for 5 min; centrifuge at 12,000× *g* 4 °C for 5 min, take 800 μL of the supernatant, add 200 μL of chloroform, shake and mix well, and leave at room temperature for 5 min; centrifuge at 12,000× *g* 4 °C for 15 min; take the supernatant, add 500 μL of isopropanol and mix well, and leave at room temperature for 10 min; centrifuge at 12,000× *g* 4 °C for 10 min. After removing the supernatant, add 500 μL of isopropanol, mix thoroughly, and leave for 10 min at room temperature. Centrifuge at 12,000× *g* for 10 min at 4 °C, discard the supernatant, add 1 mL of 75% ethanol, mix well, centrifuge at 7500× *g* for 5 min at 4 °C, discard the supernatant, and dry the precipitate at room temperature. Add RNase-free water to dissolve the precipitate. 

An ultraviolet spectrophotometer was used to determine the concentration of RNA. The concentration of the RNA sample was adjusted with RNase-free water.

#### 4.9.2. cDNA Synthesis

Reverse transcription was used (refer to the UltraStart SYBR Green qPCR Master Mix Reverse Transcription Kit instruction manual). The reverse transcription system is shown in Table 3. After the system was made, all of the reagents underwent liquid sedimentation, 25 °C reaction for 10 min, 55 °C reaction for 15 min, and 85 °C response for 5 min. After denaturing the template–primer mixture, the reaction tube was immediately put on ice to terminate the reaction. Then, 80 μL of DEPC water was added for dilution to obtain cDNA.

#### 4.9.3. qRT-PCR Assay

The qRT-PCR assay was performed according to the instructions for the StormstarSybrGreen qPCR Master Mix kit. A 20 μL system was used for the assay, and the assay system is shown in Table 4.

#### 4.9.4. Primer Design and Synthesis

Primers were designed with Oligo7 software, and species-specific primer matching was performed using the NCBI Primer-Blast system. Primers were synthesized by the Chengdu Branch of Bioengineering (Shanghai) Co., Ltd., (Shanghai, China). The primer sequence information is shown in Table 5.

#### 4.9.5. Data Processing

Data results were analyzed using the 2^−ΔΔCT^ method.

### 4.10. Immunohistochemistry Analysis

Protein expression levels were detected by immunohistochemistry. First, paraffin was removed and sliced, before being hydrated and dewaxed. Antigen repair, blocking, containment, incubation of primary and secondary antibodies, DAB color development, and hematoxylin re-staining were carried out, and gradient alcohol dehydration was followed by xylene transparency and neutral resin adhesive sealing of the film. The positive expression of the target was observed under a microscope.

### 4.11. Statistical Analysis

The data in this study were analyzed and plotted using GraphPad Prism 9.0 [31]. Significant differences between groups were tested using a one-way analysis of variance (ANOVA). *p* < 0.05 indicates statistical significance.

## Figures and Tables

**Table 1:** Instruments and reagents.

Name	CAS No./Goods No.	Batch Number	Manufacturer	Source
Yihong Y	E4009	MKCG9611	Sigma	Burlington, MA, USA
Crea	CH0101053	0623021	Mike’s Biotechnology Ltd.	Chengdu, China
Urea	CH0101051	1122071	Mike’s Biotechnology Ltd.	Chengdu, China
DAB colour development kit	ZLI-9018	234030103	Beijing Zhongshan Jinqiao Biotechnology Co., Ltd.	Beijing, China
Neutral resin adhesive	IH0265	0426A21	Beijing Leigen Biotechnology Co., Ltd.	Beijing, China
RNAiso Plus	9109	/	Takara	Shiga, Japan
UltraStart SYBR Green qPCR Master MixcDNA Synthesis Kit	A502-01	/	Rongwei Gene Biotechnology Co., Ltd.	Chengdu, China
StormstarSybrGreen qPCR Master Mix	DBI-2143	/	DBI Bioscience	Hennigsdorf, Germany
Primer synthesis	/	/	Shanghai Shenggong Biotechnology (Chengdu Synthesis Department)	Chengdu, China
Anti-TGF beta 1 antibodies	EPR21143	1047656-29	Abcam	Cambridge, UK
Anti-SMAD3 Antibody	BM3919	24F085504E08	BOSTER	Wuhan, China
Phospho-Smad3 (Ser213) Polyclonal Antibody	AB_2816415	3AB82A46	Invitrogen	Carlsbad, CA, USA
Histochemistry secondary antibody kit (rabbit)	PV-9001	2228G1129	Beijing Zhongshan Jinqiao Biotechnology Co., Ltd.	Beijing, China
DNase/RNase-Free Water	RT-121	/	Tiangen Biochemical Technology (Beijing) Co., Ltd.	Beijing, China

**Table 2:** HPLC elution conditions.

Time (min)	A (Acetonitrile)	B (0.1% Phosphoric Acid)
0	6	94
8	6	94
16	12	88
24	18	82
32	24	76
40	30	70
60	36	64

**Table 3:** Reverse transcription systems.

Name	Volumetric
10 pg-5 μg Total RNA or 10 pg-500 ng mRNA*3	As Required
DEPC-treated Water	up to 13 μL
5 × Reaction Mix*4	4 μL
Supreme Enzyme Mix	3 μL
Total Volume	20 μL

**Table 4:** RT-PCR online assay reaction system.

Name	Volumetric (μL)
PCR Forward Primer (10 μM)	0.5
cDNA Template	1
PCR Reverse Primer (10 μM)	0.5
DNase/RNase-Free Water	8
StormstarSybrGreen qPCR Master Mix	10
Total	20

**Table 5:** Primer sequence information.

ID	Primer Name	Sequences (5′ to 3′)	Tm (°C)	Product Size (Bp)
NM_008084	Mice GAPDH: F	AGGTCGGTGTGAACGGATTTG	59.1	95
	Mice GAPDH: R	GGGGTCGTTGATGGCAACA		
NM_011577	Mice TGF-β1: F	CCACCTGCAAGACCATCGAC	59.1	91
	Mice TGF-β1: R	CTGGCGAGCCTTAGTTTGGAC		
NM_016769	Mice Smad3: F	AGGGGCTCCCTCACGTTATC	59.1	77
	Mice Smad3: R	CATGGCCCGTAATTCATGGTG		
NM_007392	Mice α-SMA: F	ACTGAGCGTGGCTATTCCTTC	59.1	104
	Mice α-SMA: R	TTTCGTGGATGCCCGCTGA		

## Data Availability

The data that support the findings of this study are available from the corresponding author upon reasonable request.

## Abbreviations

The following abbreviations are used in this manuscript:

CKD	Chronic kidney disease
EC	*Eucommiae cortex*
ECF	EC ferment
ECM	Extracellular matrix
UUO	Unilateral ureteral obstruction
Pin	Pinoresinol
DA	Dehydrodiconiferyl alcohol
HPLC	High-performance liquid chromatography
OD	Optical density
CMC-Na	Carboxymethylcellulose sodium
ANOVA	Analysis of variance
